# Innate and adaptive immune evasion by dengue virus

**DOI:** 10.3389/fcimb.2022.1004608

**Published:** 2022-09-16

**Authors:** Michelle Felicia Lee, Guan Zhong Voon, Hui Xuan Lim, Mun Lok Chua, Chit Laa Poh

**Affiliations:** Centre for Virus and Vaccine Research, School of Medical and Life Sciences, Sunway University, Jalan Universiti, Bandar Sunway, Malaysia

**Keywords:** dengue virus, innate immunity, adaptive immunity, immune evasion, interferon

## Abstract

Dengue is a mosquito-borne disease which causes significant public health concerns in tropical and subtropical countries. Dengue virus (DENV) has evolved various strategies to manipulate the innate immune responses of the host such as ‘hiding’ in the ultrastructure of the host, interfering with the signaling pathway through RNA modifications, inhibiting type 1 IFN production, as well as inhibiting STAT1 phosphorylation. DENV is also able to evade the adaptive immune responses of the host through antigenic variation, antigen-dependent enhancement (ADE), partial maturation of prM proteins, and inhibition of antigen presentation. miRNAs are important regulators of both innate and adaptive immunity and they have been shown to play important roles in DENV replication and pathogenesis. This makes them suitable candidates for the development of anti-dengue therapeutics. This review discusses the various strategies employed by DENV to evade innate and adaptive immunity. The role of miRNAs and DENV non-structural proteins (NS) are promising targets for the development of anti-dengue therapeutics.

## Introduction

Dengue virus (DENV) is the most prevalent mosquito-borne pathogen that is carried by *Aedes* mosquitoes, mainly *Aedes aegypti* and *Aedes albopictus* (Murray et al., 2013). Dengue fever is common in Southeast Asia, the Western Pacific Islands, Latin America and Africa and there were approximately 390 million DENV infections being reported annually, of which 96 million were manifested clinically (Bhatt et al., 2013). DENV is a spherical 40 – 60 nm *Flavivirus* consisting of four serotypes (DENV-1, DENV-2, DENV-3 and DENV-4) from the *Flaviviridae* family. It has a positive-sense, single-stranded RNA genome of approximately 11 kb. Infection with any of the four serotypes can cause dengue fever which can rapidly develop into severe dengue including dengue hemorrhagic fever (DHF) and dengue shock syndrome (DSS) in individuals who had prior infections with heterologous DENV serotypes (Martina et al., 2009). Severe dengue is life-threatening due to the cause of excessive plasma leakage, leading to shock or, in certain cases, excessive blood loss and organ failures (Rajapakse, 2011).

Increased levels of various cytokines and chemokines such as interferon-gamma (IFN-Ɣ), granulocyte macrophage colony-stimulating factor (GM-CSF), interleukin-10 (IL-10), macrophage inflammatory protein-1 beta (MIP-1β), interleukin-1 beta (IL-1β), interleukin-8 (IL-8), tumor necrosis factor alpha (TNF-α), IFN-Ɣ -inducible protein 10 (IP-10), and monocyte chemoattractant protein-1 (MCP-1) have been associated with the progression of severe dengue (Patro et al., 2019). These cytokines are mainly secreted by monocytes. Dengue patients who developed DHF had increased levels of inflammatory C-reactive protein and inflammatory lipid enzyme mediator sPLA2 during the early stages of infections (Jeewandara et al., 2017; Vuong et al., 2020). IL-10 is a potent immunosuppressive cytokine that was strongly linked to the emergence of DHF when compared to other increased cytokines (Dayarathna et al., 2020).

It has been revealed that non-structural protein 1 (NS1) mediates pathogenesis through multiple pathways. NS1 binding to toll-like receptor 4 (TLR4) could trigger cytokine release from innate immune cells and directly contribute to vascular leak by disrupting the endothelial glycocalyx (Modhiran et al., 2015; Glasner et al., 2018) and causing endothelial apoptosis (Avirutnan et al., 2006). NS1 also induced mast cell and platelets to secrete mediators such as histamine, platelet-activating factor, and leukotrienes, resulting in inflammation and vascular leak (Glasner et al., 2018). NS1 induced complement activation by increasing C5a production, leading to increased vascular leakage and pathogenesis (Avirutnan et al., 2006). NS1 is a key target of the humoral immune response. A monoclonal antibody 2B7, which was specific for NS1 was shown to protect against NS1-mediated vascular leak and endothelial dysfunction (Biering et al., 2021). The 1G5.3 antibody has been shown to inhibit NS1-mediated cell permeability, reduced viremia and improved survival in a murine model (Modhiran et al., 2021).

Humoral and cellular immunity are important components of the adaptive immune response to DENV infections. In humoral immunity, neutralizing antibodies play an important role in limiting the spread of DENV infections. It was speculated that antibodies protect against DENV infections by complement-mediated lysis of DENV or DENV-infected cells, direct neutralization of receptor binding, antibody-dependent cytotoxicity, and Fc-receptor dependent viral clearance. Most of these neutralizing antibodies recognize the envelope (E) and pre-membrane (prM) proteins (Vázquez et al., 2002; Diamond, 2003). On the other hand, DENV infections also induce virus-specific cytotoxic T lymphocytes (CTLs) which recognize DENV-infected cells. However, DENV-induced humoral and cellular immune responses are also associated with the development of severe disease manifestations such as DHF and DSS in seropositive patients (Ye et al., 2013).

The DENV genome encodes the production of 3 structural proteins and 7 non-structural (NS) proteins. The structural proteins include the capsid (C) (11 kDa), pre-membrane (prM) (21 kDa) and envelope protein (E) (53 kDa) whilst the non-structural proteins include NS-1, NS-2A, NS2-B, NS-3, NS-4B and NS-5 (Bäck and Lundkvist, 2013). The nucleocapsid is formed when the DENV RNA genome is enclosed by the capsid protein. PrM and E protein copies are attached to the host-cell generated lipid bilayer that surrounds the nucleocapsid. The E protein consists of domains I, II and III (Rodenhuis-Zybert et al., 2010). The prM and E proteins are the major targets for immune system of the host to elicit the production of neutralizing antibodies against the virus (Whitehead et al., 2007). The 5’ UTR controls gene expression in regulating mRNA stability, localization, and translational efficiency, while the 3’ UTR contains a number of conserved RNA structures essential for viral replication (Ng et al., 2017).

However, classical targets for the development of antivirals include NS2B-NS3 protease, NS3 helicase, NS4B, and NS5 proteins. The NS3 protein has various enzymatic activities including nucleoside triphosphatase (NTPase), serine protease, helicase, and 5’-RNA triphosphatase activities whereas the NS5 protein has RNA-dependent RNA polymerase (RdRp) and methyltransferase (MTase) activities (Zou et al., 2011; Apte-Sengupta et al., 2014; Luo et al., 2015; Obi et al., 2021).

## Innate immune response to DENV infection

Dengue virus is injected subcutaneously through the proboscis of Aedes aegypti and infects Langerhans cells (LCs) which are tissue-resident dendritic cells found in the epidermis of the skin. Infected Langerhans cells migrate through the lymphatic system to the regional lymph nodes where they present dengue virus antigens on their surface to T and B cells to trigger the adaptive immune response (King et al., 2020). The other immune cells that are involved during primary DENV infections are the dendritic cells (DCs), macrophages and mast cells (MCs) (Duangkhae et al., 2018). Monocytes and macrophages are the primary targets of DENV along with the dendritic cells. DENV infections would trigger the innate response *via* pattern recognition receptors (PRRs) and the secretion of cytokines such as tumor necrosis factor (TNF) and interferon alpha (IFN-α) as well as chemokines such as chemokine ligand 5 (CCL5), C-X-C motif chemokine ligand 10 (CXCL10), and C-X-C motif chemokine ligand 12 (CXCL12) *via* degranulation of the MCs, resulting in retention and activation of T cells in draining lymph nodes (DLNs) (St John, 2013). The release of tumor necrosis factor alpha (TNF-α) would also enhance the expression of E-selectin adhesion molecules on the endothelial cells, resulting in increased recruitment of monocytes to the site of infection (Shelburne et al., 2009). LCs and DCs play an important role in the uptake of antigens and migrate to DLNs to initiate the adaptive immune responses of DENV infections (Randolph et al., 2005).

When DENV enters the host, innate immune cells (dendritic cells, macrophages and monocytes) respond by using the pathogen-recognizing receptors (PRRs) to recognize pathogen-associated molecular patterns (PAMPs) (Loo and Gale, 2011). Retinoic acid-inducible gene I (RIG-I), melanoma differentiation-associated protein 5 (MDA5) and endosomal Toll-like receptor 3 (TLR3) are the PRRs involved in DENV recognition (Nasirudeen et al., 2011). The recognition of viral genomes by cytoplasmic retinoic acid-inducible gene I (RIG-I) and melanoma differentiation-associated protein 5 (MDA5) activate mitochondrial antiviral signaling (MAVS), which results in the activation of TANK-binding kinase 1 (TBK1) and IκB kinase-ϵ (IKKϵ) to phosphorylate transcription factors, interferon regulatory factor 3 (IRF3) and interferon regulatory factor 7 (IRF7), leading to the induction of type I/III IFN (Zhao, 2013). Type I and III IFNs that are induced will bind to their respective interferon-α/β receptors (IFNAR) and activate the Janus kinase/signal transducer and activator of transcription (JAK-STAT) pathway. The pathways lead to the expression of IFN-stimulated genes (ISGs) (Haller et al., 2006; Kao et al., 2018). Other than that, the importance of IFN-α/β and IFN-Ɣ in the protection of human HepG2 cells against DENV infection has been demonstrated in various experimental studies that showed how these cytokines could limit viral replications (Diamond et al., 2000).

TIR-domain-containing adapter-inducing IFNβ (TRIF) and myeloid differentiation primary response gene 88 (MyD88) are recruited as adaptor proteins as a result of endosomal toll-like receptor 3 (TLR3) and toll-like receptor 7 (TLR7) recognizing the viral genome. These adaptor proteins then activate nuclear factor-κB (NF-κB) and IRF3/IRF7 to produce type I IFN and pro-inflammatory cytokines (Bowie and Unterholzner, 2008). The increase in type-I-IFN will elevate the innate immune response by activating the complement system and the adaptive response for viral clearance.

## Innate immune evasion

### Passive evasion through ‘hiding’ in the ultrastructure of the host

The innate immune system acts as the first line of defense against viral infections which is frequently targeted by DENV for immune evasion (Morrison et al., 2012). DENV has been shown to interfere with innate immune signaling *via* different mechanisms, thus inhibiting antiviral response. Therefore, to mount a successful infection, DENV must inhibit the main innate immune response which is based on the type I IFN system. DENV possesses several intrinsic characteristics that prevent it from being detected. Vesicle packets (VPs) are microenvironments that are localized within the endoplasmic reticulum (ER) which allow the concentration of viral proteins and metabolites that are required for viral replication. These isolated spaces also act as a mechanism of evasion to exclude themselves from potential inhibitory factors in the host (Arakawa and Morita, 2019). This passive evasion mechanism allows the delayed interaction between pathogen recognition receptors (PRRs) and pathogen-associated molecular patterns (PAMPs).

### Interference of signaling pathway through RNA modification

Upon viral replication in the isolated spaces of VPs, the RNA molecules possess the ability to interfere with the RLR-dependent signaling. RLR-signalling is the recognition pathway involving RIG-I-like receptors (RLR) for recognizing PAMPs of DENV and a disruption of this pathway leads to blocking PRR response. 2’-O methylation and partial degradation of viral RNA (vRNA) by host factors are two modification strategies that could occur to the vRNA, allowing it to prevent detection by the innate immune system (Tremblay et al., 2019). NS5 contains a domain that has methyltransferase (MTase) activity which is responsible for catalyzing the methylation that occurs on the 2’-OH position of the first nucleotide which allows the vRNA to camouflage itself as cellular mRNA (Potisopon et al., 2014). The 2’-O methylation is a common evasion strategy found in viruses and is pivotal in affecting coronavirus detection in the host (Paramasivam, 2020). Partial degradation of the vRNAs by host nucleases also acts as an evasion mechanism as incomplete degradation results in subgenomic flavivirus RNA (sfRNA) which are likely to be resistant to further degradation. The sfRNA is derived from the stalling of 5’-3’exonuclease 1 (XRN1), causing it to malfunction and prematurely cut the vRNA (Clarke et al., 2015). With reference to other flaviviruses, the accumulation of sfRNA can contribute to help the virus to circumvent the antiviral signaling pathways (Schuessler et al., 2012; Donald et al., 2016). A previous study has reported the accumulation of sfRNAs in DENV that could inhibit tripartite motif containing 25 (TRIM25) deubiquitylation by ubiquitin carboxyl-terminal hydrolase 15 (USP15) (Manokaran et al., 2015). This prevented the activation of RIG-I receptors and subsequent interactions with the mitochondrial antiviral signaling (MAVS) adaptor which led to the hijacking of IFN signaling (Okamoto et al., 2018). The 5′ and 3′ untranslated regions (UTRs) are critical for viral genome replication and translation (Ng et al., 2017). The 3’UTR induced pathogenicity by contributing to innate immune evasion of host cells. Secondary structures in the 3′ UTR have been shown to stabilize viral RNA and provide resistance to degradation by cellular RNase and promote the accumulation of sub-genomic RNAs (Funk et al., 2010).

### Inhibition of type I IFN production

**Figure 1:**
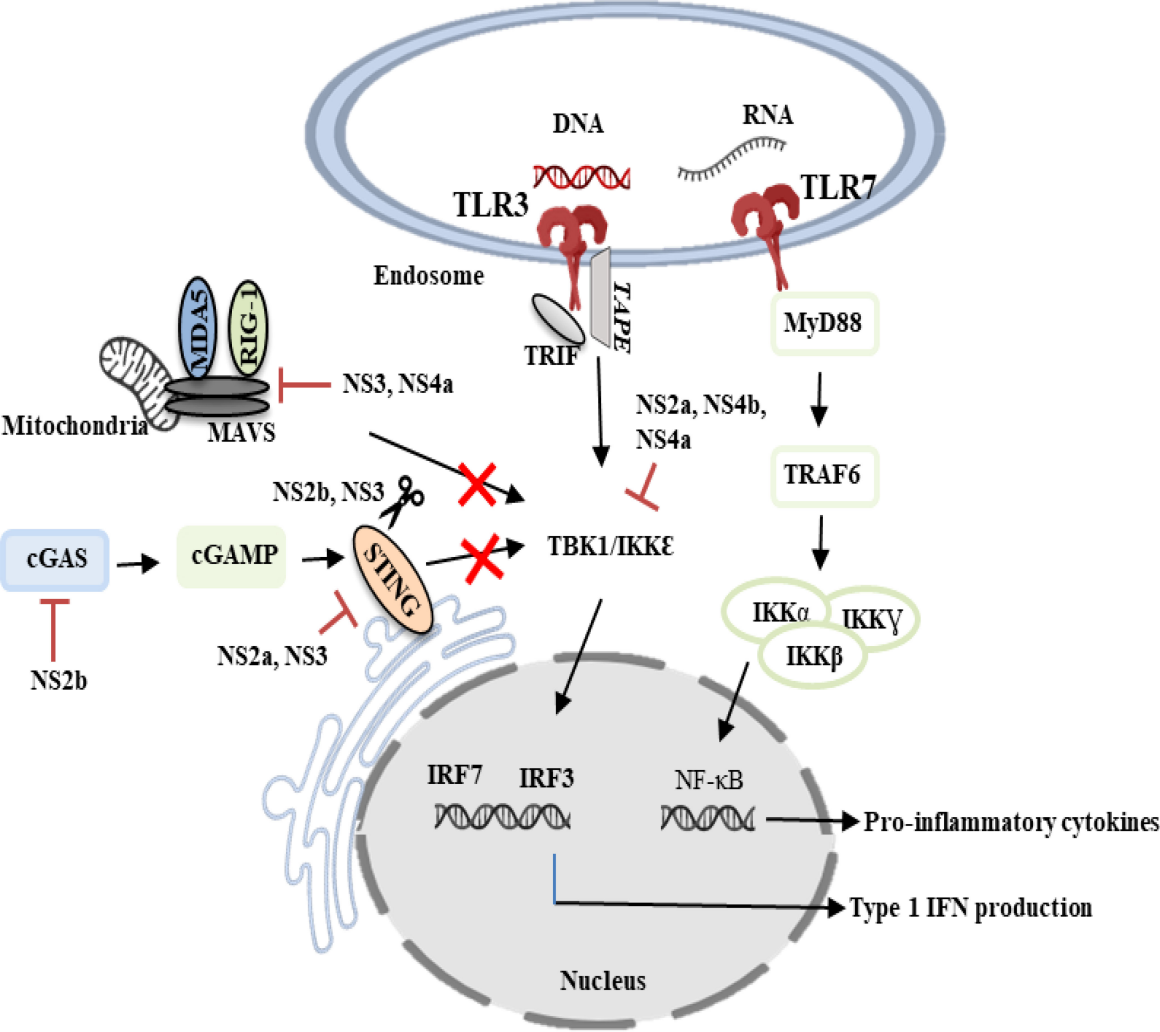
DENV evasion of innate immune responses. DENV non-structural proteins such as NS2a, NS2b, NS3, NS4a and NS4b are known to modulate innate immunity by counteracting the type I IFN-mediated antiviral response. TLR3, Toll-like receptor 3; TLR7, Toll-like receptor 7; NS2a, Non-structural 2a protein; NS2b, Non-structural 2b protein; NS3, Non-structural 3 protein; NS4a, Non-structural 4a protein; NS4b, Non-structural 4b protein; IRF3, Interferon regulatory factor 7; IRF7, Interferon regulatory factor 7; cGAS, Cyclic GMP-AMP synthase; cGAMP, Cyclic GMP-AMP; IKKα, IκB kinase-alpha; IKKβ, IκB kinase-beta; IKKƔ, IκB kinase-gamma; TRAF6, Tumor necrosis factor receptor-associated factor 6; MAVS, Mitochondrial antiviral signaling; MyD88, Myeloid differentiation primary response gene 88; TBK1/IKKϵ, TANK-binding kinase 1/IκB kinase-epsilon; RIG-1, Retinoic acid-inducible gene 1; MDA5, Melanoma differentiation-associated protein 5; TRIF, TIR-domain-containing adapter inducing IFN-β.

DENV is known as a weak inducer of type I IFN response as it has evolved multiple strategies to antagonize the host IFN system (Wu et al., 2013). The NS3 protein of DENV inhibited the translocation of RIG-I to the adaptor protein MAVS, which is found in the inner membrane of mitochondria by interacting with the chaperone protein 14-3-3ϵ (Chan and Gack, 2016). NS4a inhibited the interaction of RIG-I with the adaptor protein MAVS by binding to the N-terminal CARD-like domain and C-terminal transmembrane domain of MAVS, resulting in the suppression of IRF3 activation and IFN production (He et al., 2016). NS2a and NS4b from DENV1, 2, 4, and NS4a from DENV1 were shown to abrogate IFN-β production by blocking the RIG-I/MAVS signaling pathway and preventing phosphorylation of TBK1/IRF (Dalrymple et al., 2015). NS2b inhibited type I IFN production by targeting cyclic GMP-AMP synthase (cGAS) for lysosomal degradation and preventing mitochondrial DNA sensing (Aguirre et al., 2017). NS2b/3 protease blocked IFN production and subverted the host’s innate immunity by cleaving STING (Yu et al., 2012). NS2A and NS3 have been shown to impair RIG-I and TLR-3 signaling pathways by degrading STING and IRF3 (Castillo Ramirez and Urcuqui-Inchima, 2015). By inactivating the mitochondrial fission factor dynamin-related protein 1, NS4b induced mitochondrial elongation, resulting in altered mitochondria-associated membranes (MAMs), increased DENV replication, and decreased IFN production (Barbier et al., 2017). The involvement of DENV non-structural proteins in the evasion of innate immune response is illustrated in **Figure 1**.

### Inhibition of type I IFN signaling

**Figure 2:**
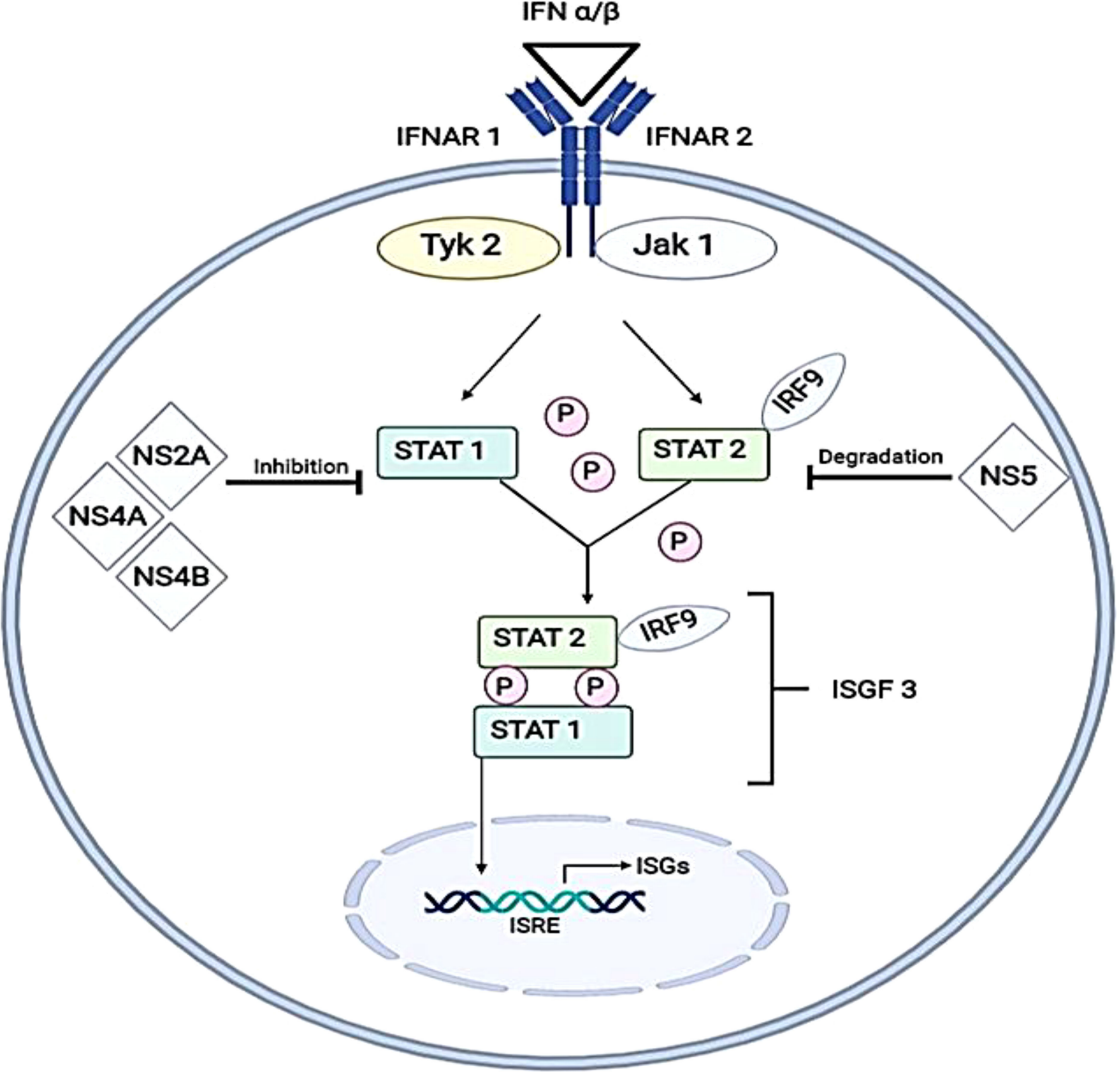
Inhibition of IFN α/β (type I IFN) signaling pathways by DENV virus. The pathway of formation for IFN-stimulated gene complex 3 (ISGF3) before translocating into the nucleus to induce IFN-stimulated genes by binding with IFN-stimulated response element (ISRE) is illustrated. DENV antagonizes this pathway by either inhibiting the active phosphorylation of STAT1 (by NS2A, NS4A and NS4B) or by direct degradation of STAT2 (by NS5). This illustrates mechanisms will occur in the absence of enhancing antibodies. IFNAR1, Interferon alpha and beta receptor subunit 1; IFNAR2, Interferon alpha and beta receptor subunit 2; Tyk2, Tyrosine kinase 2; JAK1, Janus kinase 1; IRF9, Interferon regulatory factor 9; NS2A, Non-structural 2A protein; NS4A, Non-structural 4A protein; NS4B, Non-structural 4B protein; NS5, Non-structural 5 protein; STAT1, Signal transducer and activator of transcription 1; STAT2, Signal transducer and activator of transcription 2; ISGs, Interferon-stimulated genes.

Type I IFN signaling induces transcription of IFN-signaling genes (ISGs) through the activation of the JAK-STAT pathway. This pathway involves type I IFN receptors (IFNARs) that are commonly present on nucleated cells, the activation of the signaling pathway is initiated through the binding of IFN α/β. This activates Janus kinase 1 (JAK1) and tyrosine kinase (Tyk2), which phosphorylate signal transducer and activator of transcription 1 (STAT1) and signal transducer and activator of transcription 2 (STAT2). Through these cascades of events, the phosphorylated STAT1 and STAT2 will associate with interferon regulatory factor 9 (IRF9) to form a transcription factor (IFN-stimulated gene factor 3) (ISGF3) (Fink and Grandvaux, 2013) which binds to an IFN-stimulated response element (ISRE) to turn on the transcription of ISGs (**Figure 2** **)**.

During ADE, engagement of FcR by DENV–antibody immune complexes and entry of immune complexes into target cells resulted in downregulation of expression of TLR-3, -4 and -7 (Kulkarni, 2020). Suppression of retinoic acid-inducible gene 1 (RIG-1) and melanoma differentiation-associated protein 5 (MDA-5) signaling were also observed in DENV-ADE-infected K562 cells, resulting in the inhibition of type I IFN production and IFN-mediated antiviral responses (Huang et al., 2016). Hence, type I IFN signaling would occur in the absence of enhancing antibodies.

The inhibition of the IFN signalling pathway has been reported in several publications involving human or non-human primate cells infected with DENVs and led to decreased type I IFN signalling through blocking signal transduction for phosphorylation of STAT1 or degradation of STAT2 proteins (Muñoz-Jordán et al., 2003; Jones et al., 2005; Ashour et al., 2009). Studies involving human K562 cells (Jones et al., 2005) and A549 cells (Muñoz-Jordán et al., 2003) expressing dengue replicons had shown an overall decrease of STAT 1 phosphorylation with lower STAT2 levels. On the other hand, a study using non-human primate cells (VERO cells) had shown a decrease in STAT2 levels when infected with either DENV-1 or 2 (Ashour et al., 2009).

The products of ISGs have been reported to be involved in antiviral immune response upon infection of viruses and are also pivotal in establishing the adaptive immune response which is responsible for the removal of the infection (Boo and Yang, 2010). The importance of this pathway can be further illustrated through an experiment to quantify the amount of viral RNA present in mice when comparing STAT1, STAT2 knockout mice to the wild-type mice. The results presented showed that mice with single-deficient STAT1 or 2 possessed up to 100 folds more viral RNA after 72 hours of infection (Perry et al., 2011). This indicates the importance of type I IFN response in preventing DENV RNA replication.

### Inhibition of STAT 1 Phosphorylation by NS2A, NS4A and NS4B

NS2A, NS4A and NS4B can interfere with the type I IFN response by inhibiting the upstream phosphorylation of STAT1. These non-structural proteins, NS2A, NS4A and NS4B are associated with the cellular membrane and each has its own respective role in viral replication and maturation. NS2A has been proven to have a vital role in viral assembly (Leung et al., 2008) and for NS4A and NS4B, they have been shown to co-localize with double-stranded RNA (dsRNA) and the structural protein E, suggesting that these proteins are associated with replication complex of the virus (Miller et al., 2007).

It is interesting to know that even though these non-structural proteins inhibit ISG production in the JAK-STAT pathway, only NS4B has an effect that is potent enough to have an antagonistic effect on the pathway. Otherwise, the effect of the STAT1 inhibition can be recapitulated and points to the presence of an interplay between the three viral proteins in inhibiting IFN type I response (Muñoz-Jordán et al., 2003; Munoz-Jordán et al., 2005).

A dysfunctional innate immune response can lead to the manifestation of severe dengue symptoms due to the inhibition of both IFN production and IFN signaling pathways, as well as increased secretion of inflammatory cytokines, lipid mediators and chemokines (Malavige et al., 2020).

### Immune evasion from complement response

Evasion of the complement response by DENV is modulated by secretion of the NS1 protein. NS1 inhibits the activation of the classical and lectin pathways of the complement system by binding to the complement protein C4 and activating the protease C1s, leading to the cleavage of C4 to C4b and reduces the deposition of C4b on the surface (Avirutnan et al., 2010). Avirutnan et al. (2011) demonstrated that NS1 recruited C4b binding protein, which could suppress complement pathways by inhibiting C4b activity (Avirutnan et al., 2011). Other than that, NS1 prevented the formation of membrane attack complex by forming the NS1-VN complex with the complement regulator vitronectin (VN). This interaction has been observed both *in vitro* and in DENV-infected patients (Conde et al., 2016). Lastly, NS1 has shown ability to evade from complement response by competitively binding to mannose-binding lectin (MBL) and preventing DENV recognition by MBL, thereby protecting the virus from neutralization (Thiemmeca et al., 2016).

### miRNAs that inhibited DENV replication by regulating host IFN system

miRNA is a small single-stranded non-coding RNA molecule with approximately 22 nucleotides that functions in post-transcriptional regulation of gene expression (Ambros, 2004). miRNA plays a pivotal role in the regulation of viral replication (Umbach and Cullen, 2009). Upregulation of miRNA-30e* in HeLa and U937 cells upon DENV infection restored type I IFN production and could suppress DENV replication through targeting Iκβα and subsequent activation of NF-κB signaling (Zhu et al., 2014). Overexpression of Let-7c was shown to inhibit the replication of DENV-2 and DENV-4 in human hepatoma Huh-7 cells through the modulation of host factors such as heme oxygenase-1 (HO-1) and BTB domain and CNC homolog 1 (BACH1). HO-1 has been found to reduce DENV replication by suppressing NS2B/NS3 protease activity, resulting in the stimulation of antiviral IFN responses (Tseng et al., 2016). Additionally, miRNA-155 has been shown to block DENV replication by specifically targeting BACH1, which induced HO-1-mediated suppression of NS2B/NS3 and enhanced antiviral IFN responses (Su et al., 2020). miRNA-34 increased the production of type I IFN and ISG expression by inhibiting the Wnt signaling, resulting in the inhibition of viral replication (Smith et al., 2017). The expression of the SIAH E3 ubiquitin protein ligase 1 (SIAH1) was found to be downregulated by miRNA-424, which was then shown to impede DENV replication. By binding to and ubiquitinating the adaptor protein MyD88, inhibition of SIAH1 expression resulted in promoting MyD88-mediated NF-κB signaling (Murphy Schafer et al., 2020).

## Evasion of adaptive immune responses by DENV

**Figure 3:**
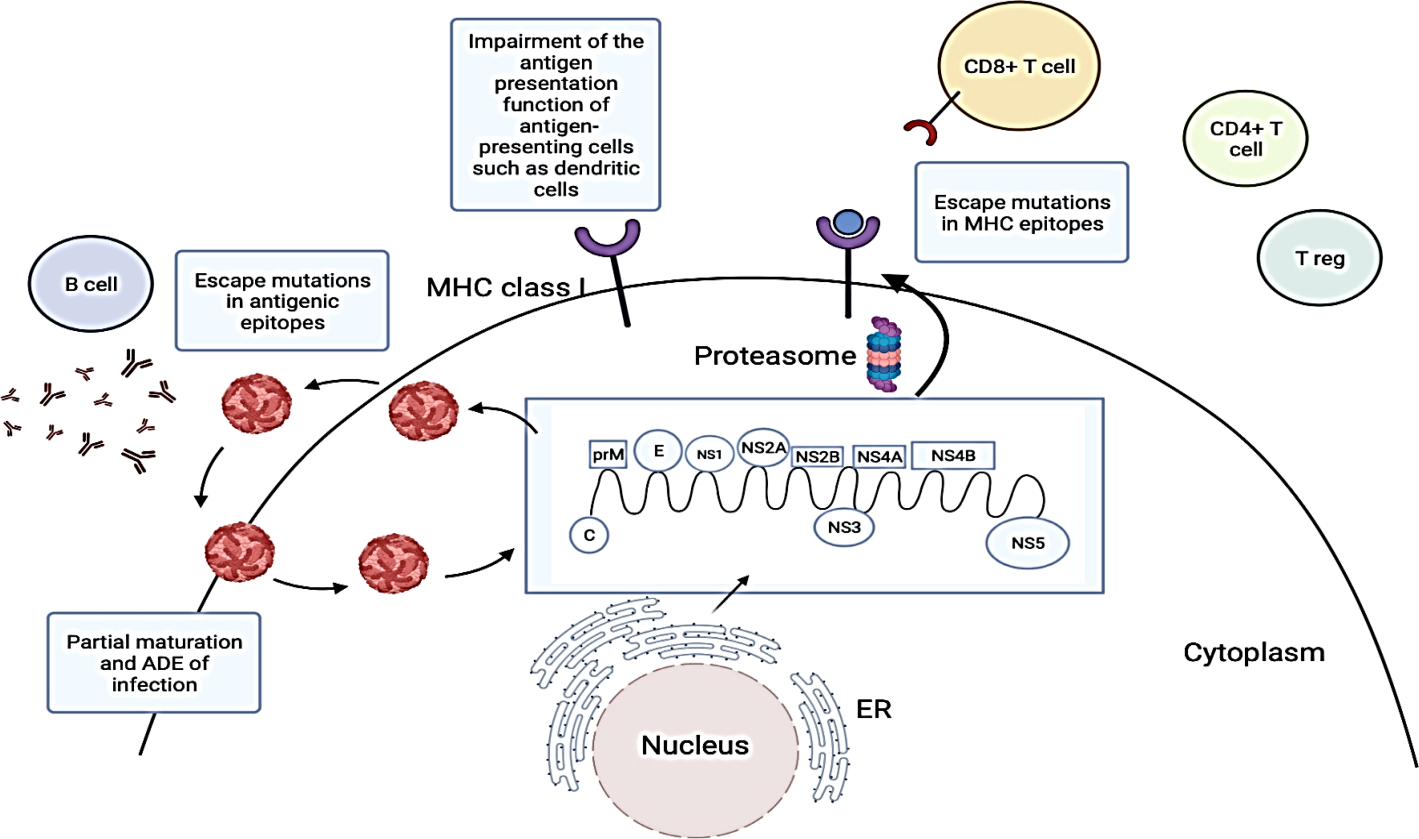
Strategies employed by dengue virus (DENV) to evade adaptive immune responses. DENV viral proteins are produced by translating the DENV RNA genome. DENV structural proteins and progeny RNA genomes are then assembled into new viral particles which are released *via* the secretory pathway. These viral proteins are usually degraded by proteasomes and peptides associated with major histocompatibility complex (MHC) class I molecules are presented on the surface of DENV-infected cells to CD8+ T cells. However, DENV may undergo antigenic mutations to evade recognition by neutralizing antibodies and T cells (Chareonsirisuthigul et al., 2007; Martinez et al., 2020). They can also evade adaptive immune responses by promoting viral entry into cells through pre-existing antibodies and inhibiting antigen presentations by antigen-presenting cells. The figure has been created with Biorender.com (ON, Canada). ADE, Antibody dependent enhancement; C, Capsid protein; E, Envelope protein; ER, Endoplasmic reticulum; prM, Pre-membrane protein; NS1, Non-structural 1 protein; NS2A, Non-structural 2A protein; NS2B, Non-structural 2B protein; NS3, Non-structural 3 protein; NS4A, Non-structural 4A protein; NS4B, Non-structural 4B protein; NS5, Non-structural 5 protein.miRNAs as potential therapeutics for dengue infections.

Apart from the innate immune system, dengue virus (DENV) also utilises various strategies to evade the adaptive immune system. Adaptive immunity is activated when the innate immune response fails to eliminate an infection whereby antigen and activated antigen-presenting cells (APCs) are transported to the draining lymphoid tissues (Janeway, 2001). The adaptive immune system is made up of the humoral immune response (mediated by B cells and neutralizing antibodies) and the cell-mediated immune response (mediated by CD4^+^ and CD8^+^ T cells). Both arms of the adaptive immune system play important roles in DENV disease pathogenesis **(** **Figure 3** **)** (Ye et al., 2013).

### Humoral immune response

The humoral immune response plays an important role in controlling DENV infection and dissemination. Neutralizing antibodies (nAbs) play a central role in the humoral immune system by preventing and clearing an infection. DENV infection induces the production of high titres of nAbs which provide long-term immunity to the DENV serotype during a primary infection and short-term immunity to a heterologous DENV serotype during a secondary infection. nAbs function to prevent DENV entry into cells by recognizing conformational epitopes, glycans, and specific regions of the envelope or capsid proteins. However, DENV-induced humoral immune responses are also associated with the development of severe disease manifestations such as DHF and DSS (Chiappelli et al., 2014). Therefore, this suggests that dengue viruses have evolved various strategies to modulate the host humoral immune responses.

### Antigenic variation to evade recognition by neutralizing antibodies

The rapid evolution of RNA viruses occurred due to the RNA polymerase which is prone to errors during viral RNA replication. The build-up of mutations in the viral genome and selection pressure of the host immunity might lead to changes in the viral proteins (Ye et al., 2013). Apart from that, RNA viruses also contain low fidelity RNA-dependent RNA polymerases, which generate viral quasispecies that contain random mutations throughout its viral genome. These viral quasispecies contain distinct antigenic epitopes which enable evasions of recognition by host neutralizing antibodies (Diamond, 2003; Ye et al., 2013). Mutations that caused changes to the amino acid residues in domain III of the DENV E protein have been reported to assist in viral evasion from host neutralizing antibodies during DENV infections (Lin et al., 1994; Lok et al., 2001). A previous study reported that antibody-escape DENV-1-related variants isolated from the sera of DENV-infected patients replicated to higher titres than the circulating DENV-1 wild type (Chua et al., 2006). Genetic variations between the E proteins of DENV-4 genotypic variants were also reported to cause a difference in their maturation status, glycosylation pattern, growth kinetics, and foci morphology. In addition, this study also reported that infection or vaccination with a particular DENV-4 variant induced variable levels of neutralizing antibodies towards other DENV-4 variants (Gallichotte et al., 2018). Another study investigated the impact of genetic variations between the prM and E proteins of DENV-2 genotypic variants on the responses of human neutralizing antibodies. It was found that certain DENV-2 strains were more susceptible to neutralizing antibodies as compared to other DENV-2 strains (Martinez et al., 2020). Therefore, these data suggested that antigenic variations might be an immune evasion strategy employed by DENV which led to higher levels of DENV infections in hosts.

### Antibody-dependent enhancement (ADE) of infection

The humoral immune system may also be directly subverted by DENV to facilitate infections. DENV may use immune complexes that contain infectious viruses to promote the spread of infections *via* the lymphatic system through a mechanism known as antibody-dependent enhancement (ADE) (Diamond, 2003). ADE plays a significant role in the pathogenesis of secondary DENV infections (Halstead, 2003). Studies have found that neutralizing antibodies elicited from the first infection bind to DENV and promote its entry into Fc receptor-bearing cells, resulting in ADE and caused more severe dengue. In addition, it also exploits these enhancing antibodies for intracellular immune evasion by carrying out two mechanisms (Chareonsirisuthigul et al., 2007; Modhiran et al., 2010; Ubol et al., 2010). First, entry of DENV-antibody complexes into human monocytic cells activate the negative signalling regulators: authophagy-related 5-authophagy-related 12 (Atg5-Atg12), selective androgen-receptor modulator (SARM), dihydroxyacetone kinase (DAK), TANK. SARM and TANK function to disrupt the toll-like receptor (TLR) signalling cascade whereas Atg5-Atg12 and DAK function to inhibit the RIG-I/MDA-5 signalling cascade. This disabled the production of type I IFN, resulting in suppression of antiviral responses. Second, ADE reduced the production of inflammatory mediators such as interleukin-12 (IL-12) and IFN-Ɣ, but the production of immunosuppressive mediators such as interleukin-10 (IL-10) which induced the expression of the suppressor of cytokine signalling 3 (SOCS-3) gene, resulting in inhibition of the JAK-STAT pathway (Ye et al., 2013). This resulted in an increase in DENV viral loads, leading to severe disease manifestations. Apart from that, IL-10 could also suppress T cell activation, degranulation and cytokine production. The production of cytokines such as B-cell activating factor (BAFF) and proliferation-inducing ligand (APRIL) which played a role in stimulating the transformation of resting B cells into plasma cells might also be responsible for further production of DENV-specific antibodies, leading to ADE (Malavige et al., 2020).

### Partial maturation

It is a requirement to cleave the prM protein for the activation of DENV infectivity. However, studies have shown that the process of maturation and cleavage of prM for DENV was inefficient (van der Schaar et al., 2007; Zybert et al., 2008). Incomplete prM cleavage was associated with the presence of an acidic residue at position P3 within the 13 amino acid sequence proximal to the prM protein (Keelapang et al., 2004; Junjhon et al., 2008). Studies have found that the presence of prM antibodies caused immature DENV particles to become highly infectious. The prM antibodies facilitated viral entry into cells followed by virus maturation catalysed by endosomal furin, and activation of its membrane-fusion machinery (Rodenhuis-Zybert et al., 2010; Rodenhuis-Zybert et al., 2011). The prM antibodies could enhance the infectivity of prM-containing DENV particles due to interactions with Fc receptors expressed on immune cells and heat shock protein 60 which was expressed on certain Fc receptor-deficient cells (Huang et al., 2006; Rodenhuis-Zybert et al., 2010; Rodenhuis-Zybert et al., 2011). Immature DENV particles were not able to bind efficiently to cells in the absence of antibodies and thus they did not affect disease pathogenesis during primary DENV infections. However, during secondary infections, the prM-specific antibody responses would induce the infectivity of immature DENV particles, thereby increasing the number of infectious DENV virions in the blood circulation (Rodenhuis-Zybert et al., 2011). This indicated that prM-containing viruses were more prone to cause ADE in contrast to fully mature DENV particles. The findings of the infective potential of immature DENV virions in the presence of antibodies not only suggested that incomplete cleavage of prM occurred during natural DENV infections, but it is also possible that it could be a mechanism to evade humoral immune responses (Ye et al., 2013).

### Cellular immune response

Apart from humoral immunity, activation of cell-mediated immune responses are also vital for clearance of established DENV infections. The recognition of viral peptides presented by major histocompatibility complex (MHC) class I molecules on cytotoxic T lymphocytes (CTLs) is a significant event in eliminating cells which produce viral proteins during DENV infections. CTLs play a vital role in controlling viral infections, especially as a long-term immune surveillance effector that can react rapidly against the same virus during a secondary infection (Ye et al., 2013).

For example, to establish an infection, DENV requires T helper 1 (T_H_1) responses which stimulate the production of interleukin-12 (IL-12), interleukin-18 (IL-18), tumor necrosis factor (TNF), and IFN-γ. Both TNF and IFN-γ are known to be associated with DENV disease severity. However, it has also been reported that T_H_1 cells may play a role in limiting DENV pathogenesis, suggesting that the balance in induction of T helper cells is of vital importance (Rivino et al., 2013; Mathew et al., 2014; Rivino, 2016; Rivino and Lim, 2017). Studies have shown that co-culturing DENV-infected dendritic cells (DCs) and naive T helper cells resulted in the formation of T_H_1 cells which secreted IFN-γ. This process is dependent on the activation of RIG-I-like receptors (RLRs) during DENV replication. The differentiation of T_H_1 cells is induced by IL-12, a cytokine consisting of IL-12p35 and IL-12p40 subunits. However, triggering of RLRs during DENV infection did not induce the production of IL-12 as RLR-activated IRF3 inhibited IL-12p40. Type 1 IFN and interleukin-27 (IL-27) are also known to induce the differentiation of T_H_1 cells. RLR activation during DENV infection induced the secretion of both type 1 IFN and IL-27 and they acted as mediators for T_H_1 polarization, resulting in production of DHF- and DSS-associated inflammatory mediators, TNF and IFN-γ (Sprokholt et al., 2017a; Sprokholt et al., 2017b; Sprokholt et al., 2017).

Apart from T_H_1 cells, the expansion of cytotoxic CD4^+^ and CD8^+^ T cells from naive T cells was also detected during DENV infection. These cells acquire a high affinity for the primary infecting DENV serotype. The triggering of T-cell receptors (TCRs) of DENV-specific cytotoxic T cells resulted in the production of IFN-γ and an increase in the expression of CD107a on the cell surface (Gagnon et al., 1999; Tian et al., 2016). The production of IFN-γ and upregulation of CD107a expression led to lysis of DENV-infected cells and the amount of cytotoxic CD4^+^ and CD8^+^ T cells which expressed CD107a were associated with protection against DENV (Duangchinda et al., 2010; Sprokholt et al., 2017). Hence, due to the various roles played by CTLs during DENV pathogenesis, evasion of CTLs is a requirement for productive DENV viral replications (Ye et al., 2013).

### Inhibition of antigen presentation

Dendritic cells (DCs) play an important role in bridging both innate and adaptive immune responses by integrating signals from pathogen-associated microbial patterns (PAMPs) with pathogen-derived antigens to stimulate antigen-specific B cell and T cell responses. DCs carry out functions such as (a) uptake and processing of antigens, followed by presentation of the antigen-derived peptides on MHC class I molecules to CD8^+^ T cells or on MHC class II molecules to CD4^+^ T cells; (b) expressing co-stimulatory molecules for activation of T cells; and (c) secreting cytokines and chemokines to lure T cells and modulate priming of T cells (Schmid et al., 2014).

Since the survival of DCs is important for optimal T cell activation, DENV-induced apoptosis of DENV-infected DCs could inhibit the priming of adaptive immune responses. Studies have reported that non-infected bystander monocyte-derived DCs (moDCs) upregulated MHC class I and II molecules and co-stimulatory molecules such as CD80 (B7-1), CD83 and CD86 (B7-2) after exposure to DENV. However, within the same culture, DENV blocked the activation and maturation of infected moDCs (Libraty et al., 2001; Palmer et al., 2005). In another study, it was also observed that non-infected bystander monocytes, moDCs and classical DCs (cDCs) expressed higher levels of CD80 and CD86 as compared to DENV-infected cells in infected mice deficient in IFN-α/β receptor (Schmid and Harris, 2014). These observations suggested that DENV induced apoptosis in DENV-infected cells, but increased its survival in non-infected bystander cells. Additionally, these results also indicated that the blockage of activation in DENV-infected DCs by DENV might reduce the priming of CD4^+^ or CD8^+^ T cells, whereas non-infected bystander cells could still be activated (Schmid et al., 2014).

Studies have also shown that DENV infection reduced the capacity of moDCs and DCs isolated from human skin explants to prime DENV-naïve CD4^+^ T cells, suggesting that DENV-infected DCs had an impaired to activate CD4^+^ T cells (Palmer et al., 2005; Nightingale et al., 2008; Cerny et al., 2014). Others have also reported similar results that DENV-infected moDCs could stimulate CD4^+^ T cells, but with reduced T cell effector functions, such as reduced secretions of IFN-γ and TNFα (Chase et al., 2011). The impaired ability of DENV-infected moDCs to produce IFN-α and IFN-β might also explain their reduced ability to prime T cell responses (Rodriguez-Madoz et al., 2010). Therefore, inhibition of antigen presentation and functionality of DENV-infected DCs might be a viral evasion strategy to reduce T cell responses.

### Antigenic variation to evade recognition by T cell receptors

Apart from enabling viral escape from neutralizing antibodies, viral quasispecies formed during DENV infections might also facilitate evasion of the recognition by MHC molecules or T cell receptors. Viral quasispecies formed during infection might also reduce the amount of T cell epitopes that could promote effector functions during the infection (Diamond, 2003). A study by Erickson et al. (2001) revealed that the hepatitis C virus (HCV) quasispecies acquired mutations in multiple epitopes which resulted in impairment of MHC class I binding and CTL recognition (Erickson et al., 2001). For the case of DENV infections, studies have shown that complexity in the pattern of functional immune responses to heterologous DENV serotypes were highly dependent on the amino acid sequences and epitopes of the variant peptides (Bashyam et al., 2006). This might also justify the strong correlation of DHF with high cytokine levels and heterologous secondary DENV infections. It has been demonstrated that dengue-specific memory T cells have high binding affinities to dengue antigens from primary infections and poor binding affinities to dengue antigens from secondary infections caused by heterologous serotypes, resulting in inadequate immune responses. This phenomenon is known as the “original antigenic sin” (Mongkolsapaya et al., 2003).

### miRNA regulation of adaptive immunity response

Apart from regulating innate immunity, miRNAs have been widely associated with regulating adaptive immunity by modulating the survival, activation, development, and proliferation of B- and T-cells (Raisch et al., 2013).

### miRNAs regulate T-cell development, differentiation, and activation

T-cell development involves the roles of various signaling cascades which are mediated by miRNAs. It has been discovered that disruption in the biogenesis of miRNAs might cause a conditional removal of dicer in the early stage of T-cell development, resulting in a reduction of T-cell counts. Additionally, it has also been observed that this disruption might also lead to deviant cytokine production and differentiation of T-helper cells as well as reduced survival of αβ-expressing thymocytes. Poor proliferations, reductions in number, and increased apoptosis were also observed in peripheral T-cells (Koenecke and Krueger, 2018; Rodríguez-Galán et al., 2018). miRNAs such as the miRNA-181 family, miRNA-17-92 clusters, miRNA-214, miRNA-146a, miRNA-29, let-7, miRNA-125, miRNA-155, and miRNA-216 were observed to play a role in the signalling cascade downstream of T-cell activation (Luan et al., 2018). For example, miRNA-155 has been shown to play a critical role in the differentiation of CD4+ T-cells. Overexpression of miRNA-155 is associated with the differentiation of CD4+ T cells into Th1 cells and reduced expression of miRNA-155 demonstrated a bias toward cell differentiation (Baumjohann and Ansel, 2013). miRNA-155 is expressed abundantly in dendritic cells, macrophages, as well as active B and T cells (Pareek et al., 2014; Xia et al., 2018; Ekiz et al., 2019; Wang et al., 2019). A study by Su et al. (2020) reported that the expression of miRNA-155 was downregulated during DENV infection, thereby suggesting its possible role against oxidative stress in DENV-induced pathogenesis. Their results demonstrated that miRNA-155 inhibited DENV replication *via* induction of the heme oxygenase-1 (HO-1) signalling pathway and enhanced antiviral IFN responses. They also observed that the exogenous expression of miRNA-155 in the brains of ICR suckling mice which provided a protective effect against life-threatening DENV infection (Su et al., 2020). Another miRNA, miRNA-146, modulated immune responses by targeting tumor necrosis factor receptor-associated factor 6 (TRAF6) and interleukin-1 receptor-associated kinase 1 (IRAK1) of the NF-κβ signalling in activated T-cells (Saki et al., 2015). (Pu et al., 2017) demonstrated that inhibition of autophagic activity in DENV-2-infected A549 and THP-1 cells by miR-146a was due to targeting TRAF6 which resulted in a reduction of IFN-β (Pu et al., 2017). In addition, miRNAs also play an important role in regulating the function of Treg cells. Treg cells have important functions in maintaining immune cell homeostasis by preventing autoimmunity and limiting immune responses. Various miRNAs such as miRNA-10a, miRNA-146a, and miRNA-155 have been found to contribute to Treg homeostasis and functions (Hippen et al., 2018; Chandan et al., 2019).

### miRNAs regulate B-cell development, differentiation, and activation

The expression of miRNAs in B cells have been observed which implies its role in the development and maturation of B cells (Chen et al., 2018). For example, it was found that miRNA-155 suppressed the expression of c-MAF (transcription factor) and IFNγ receptor 1 in naive CD4+ T cells whereas it blocked the expression of SH-2 domain containing inositol 5’ polyphosphatase 1 (SHIP1) and PU.1 (transcription factor) in B cells. However, in the absence of miRNA-155, defective antibodies were produced and thus, impairing immune responses to antigenic stimulation. Additionally, murine models deficient in miRNA-155 were observed to show an increased number of germinal central B cells as well as reduced IgG production and maturation. The regulation of germinal central B cells was mediated by regulating PU.1 during the post-transcriptional stage (Danger et al., 2014). Apart from that, it was discovered that the expression pattern of C-Myb which plays a critical role in B cell development was inversely correlated to the expression of miRNA-150 in B cells. miRNA-150 was shown to be highly expressed in progenitor B cells whereas it was downregulated in mature B cells. High levels of miRNA-150 were necessary for the conversion of pre-B to mature B cells, so as to downregulate the expression of C-Myb for normal B cell development (Tan et al., 2009; Chandan et al., 2019). However, not much is known regarding the role of miRNAs in regulating B cell immune responses during DENV infections.

## Conclusion

In order to establish an infection, DENV must face multiple host inhibitory responses generated by both the innate and adaptive immune systems. Over the course of time, dengue viruses have developed multiple strategies to evade host immune responses. However, the specific mechanisms utilised by DENV to avoid detection and elimination by the host immune system still remains unanswered. DENV non-structural proteins are important in disabling induction of cellular IFN and signalling responses. What are the specific molecular mechanisms for the antagonism between DENV viral proteins and cellular signal mediators? Does DENV actively block the antiviral activities of IFN? Does DENV interfere with the expression of molecules such as chemokines, integrins, and receptors which are involved in the migration of immune cells? Identification of DENV immune evasion strategies and an extensive understanding of the molecular mechanisms of the viral immune evasion will facilitate the elucidation of dengue disease pathogenesis. In addition, identification of specific DENV viral proteins which trigger viral evasion could serve as potential targets for the design of novel therapeutics or vaccines to combat against dengue.

Can we prevent the immune evasion strategies employed by DENV? miRNAs have been shown to play important roles in affecting DENV replication and pathogenesis which makes them potential targets for miRNA-based antiviral therapeutics against dengue infections. The understanding of the roles played by miRNAs in important processes associated with dengue infections are necessary to fully characterize their potentials as antivirals for treatment. For example, Miravirsen, an oligonucleotide which was able to inhibit the function of miR-122a in HCV has demonstrated that miRNA-based therapeutics are promising strategies to delve into, considering that there is still a lack of effective anti-dengue therapeutics at present.

## Author contributions

ML, HL, GV, and MC wrote the manuscript. ML, GV, and HL prepared the figures. CP supervised, edited and reviewed the manuscript. All authors contributed to the article and approved the submitted version.

## Funding

The publication of this article was funded by FRGS grants (FRGS/1/2020/SKK06/SYUC/03/1) and (FRGS/1/2021/SKK0/SYUC/01/1) to CP and HL, respectively, from the Centre for Virus and Vaccine Research (CVVR), School of Medical and Life Sciences, Sunway University.

## Conflict of interest

The authors declare that the research was conducted in the absence of any commercial or financial relationships that could be construed as a potential conflict of interest.

## Publisher’s note

All claims expressed in this article are solely those of the authors and do not necessarily represent those of their affiliated organizations, or those of the publisher, the editors and the reviewers. Any product that may be evaluated in this article, or claim that may be made by its manufacturer, is not guaranteed or endorsed by the publisher.
